# Pch Genes Control Biofilm and Cell Adhesion in a Clinical Serotype O157:H7 Isolate

**DOI:** 10.3389/fmicb.2018.02829

**Published:** 2018-11-23

**Authors:** Elisa Andreozzi, Nereus W. Gunther, Erin R. Reichenberger, Luca Rotundo, Bryan J. Cottrell, Alberto Nuñez, Gaylen A. Uhlich

**Affiliations:** Molecular Characterization of Foodborne Pathogens Research Unit, Eastern Regional Research Center, Agricultural Research Service, United States Department of Agriculture, Wyndmoor, PA, United States

**Keywords:** *E. coli* O157:H7, biofilm control, cell adhesion, curli, horizontally transferred DNA regions, *pch* regulatory genes, *pchE* gene, locus of enterocyte effacement

## Abstract

In a previous study, induction of the *Escherichia coli* serotype O157:H7 SOS response decreased *csgD* expression in the clinical isolate PA20 at 30°C but strongly induced genes in the horizontally transferred-DNA regions (HTR), including many known virulence regulators. To determine the role of HTR regulators in the control of *csgD* and curli, specific regulators were plasmid-expressed in the wild-type and mutant strains of PA20 and its biofilm-forming derivative, 20R2R. At 30°C, plasmid over-expression of the O157:H7 group 3 *perC* homolog, *pchE*, strongly repressed PA20 *csgD* transcription (>7-fold) while the group 1 homologs, *pchA* and *pchB*, resulted in smaller reductions (<2.5-fold). However, SOS induction decreased rather than increased *pchE* expression (>6-fold) making group 1 *pch*, which are enhanced by the SOS response, the likely SOS-induced *csgD* repressors. Plasmid-based *pchE* over-expression also reduced 20R2R biofilm formation (>6-fold) and the curli-dependent, Congo red affinity of both PA20 and 20R2R. However, to properly appreciate the regulatory direction, expression patterns, and environmental consequences of these and other CsgD-controlled functions, a better understanding of natural *pchE* regulation will be required. The effects of HTR regulators on PA20 and 20R2R adhesion to HEp-2 cell at host temperature were also studied. Under conditions where prophage genes were not induced, curli, rather than *espA*, contributed to host cell adhesion in strain 20R2R. High levels of *pchE* expression in trans reduced curli-dependent cell adherence (>2-fold) to both 20R2R and the clinical isolate PA20, providing a host-adapting adhesion control mechanism. Expression of *pchE* was also repressed by induction of the SOS response at 37°C, providing a mechanism by which curli expression might complement EspA-dependent intimate adhesion initiated by the group1 *pch* homologs. This study has increased our understanding of the O157 *pch* genes at both host and environment temperatures, identifying *pchE* as a strong regulator of *csgD* and CsgD-dependent properties.

## Introduction

Infection with Shiga toxin-producing *Escherichia coli* (STEC) cause sporadic cases or large outbreaks of hemorrhagic colitis that can progress to the severe renal-associated syndrome, hemolytic uremic syndrome (HUS) (Tarr et al., 2005). Although Shiga toxin(s) are the most important virulence determinants, the prophage-encoded locus of enterocyte effacement (LEE) also contributes to STEC virulence (Frankel et al., 1998). In the United States, serotype O157:H7 is responsible for the highest numbers of reported STEC cases, hospitalizations, and morbidities (Scallan et al., 2011). Ruminants are the predominant STEC reservoir and fecal contamination that leads to contaminated carcasses and agricultural environments is a primary source for transmission to humans (Lim et al., 2010). A critical step in the transmission process is the transition from the reservoir GI tract to the environment where the bacterium must survive until returning to GI conditions of the host or a different reservoir. While outside of the reservoir host, formation of biofilms increases stress resistance, prolongs pathogen survival time, and increases the opportunity for reservoir to host transmission (Kumar and Anand, 1998).

In *E. coli* biofilm production, initial attachment of motile bacteria to surfaces is followed by the formation of irreversible attachments to the surface and surrounding bacteria (Kostakioti et al., 2013). This stage is dependent on amyloid-containing curli fimbriae, and the exopolysaccharide, cellulose (Zogaj et al., 2001). Curli are assembled from the CsgA subunit and CsgB nucleator proteins encoded in the *csgBAC* operon. The CsgD transcription factor controls curli formation and is encoded along with export proteins in the *csgDEFG* operon (Hammar et al., 1995; Robinson et al., 2006). Expression of *csgD* at environmental temperatures (≤30°C) utilizes the RpoS sigma factor and requires the transcription factor, *mlrA* (Brown et al., 2001). MlrA binds to sites upstream of the −35 promoter region and positively regulates *csgD* transcription in *E. coli* strains that depend on RpoS for curli expression (Brown et al., 2001; Ogasawara et al., 2010a). How MlrA is activated is currently unknown. A complex network of regulatory proteins, small RNAs, and small signaling molecules controls the *rpoS*-dependent expression of *csgD* (Gerstel and Römling, 2003; Sommerfeldt et al., 2009; Ogasawara et al., 2010a,b; Jørgensen et al., 2012). Congo red (CR) dye stains amyloid and its inclusion in agar plates allows easy identification of curli production (Zogaj et al., 2001). There is marked strain and serovar variability in the expression of curli and cellulose, CR affinity, and the production of biofilm among STEC strains (Chen et al., 2013; Uhlich et al., 2013, 2014). However, CsgD appears to retain an essential role in STEC curli and biofilm formation.

The low incidence of biofilm production in O157:H7 strains isolated from the human host suggests that biofilm structural or regulatory components may have a negative effect on viability in the human host (Biscola et al., 2011). Attenuation of biofilm properties in clinical strains generally results from genome modifications that lower *csgD* expression causing reductions in curli production (Uhlich et al., 2013). In a study of 55 clinical O157:H7 strains, 53 carried a prophage inserted in *mlrA* and two strains carried *rpoS* mutations that reduced *csgD* expression and prevented biofilm formation. Although insufficient to support biofilm formation, many of the clinical strains produced sufficient curli to bind detectable CR dye at 37°C in a *csgD*-dependent manner suggesting that reduction rather than elimination of *csgD* expression was favored (Uhlich et al., 2014). A different study confirmed that strains carrying a prophage in *mlrA* still generate considerable levels of *csgD* expression, even when curli expression is below the threshold required to produce observable CR affinity (Uhlich et al., 2016). Whether this level of *csgD* expression is utilized for controlling processes other than curli expression that are important for host colonization and disease pathogenesis is currently unknown. When LEE-generated effacing lesions are initiated, STEC require cell attachment mediated by the EspA protein, which is encoded along with other effector proteins in the LEE operon (Ebel et al., 1998; Nataro and Kaper, 1998). Transcription of *espA* depends on LEE activator, Ler, and production of EspA filaments requires a post-transcriptional mechanism allowing translation of *espABD* transcripts (Roe et al., 2003). The requirement for EspA in adhesion of O157:H7 to cultured cells has been clearly demonstrated but the effect of curli on cell adhesion is still unclear and likely varies with STEC serotype, strain genetic background, and growth conditions (Richter et al., 2014; Sharma et al., 2016; Kudva et al., 2017).

Exposure of clinical O157:H7 strains to DNA-damaging antibiotics, such as sulfamethoxazole-trimethoprim (SMX-TM), can potentiate HUS by increasing the expression of virulence genes encoded in prophage and prophage-like elements (e. g. *stx_2_* and LEE) (Herold et al., 2005; Landstorfer et al., 2014; Uhlich et al., 2018). SMX-TM can also induce prophage excision from *mlrA*, making restoration of CsgD function a possible additional consequence of antibiotic treatment in the host (Shaikh and Tarr, 2003; Uhlich et al., 2016). However, in an RNA-seq study testing SMX-TM effects on a clinical O157:H7 transcriptome, SMX-TM had a slight repressive, rather than enhancing, effect on *csgD* functions in spite of strong induction of prophage and prophage-like genes (Uhlich et al., 2018). The mechanism controlling the unexpected repressive *csgD* effects following SMX-TM exposure were not apparent but we reasoned that transcription factors differentially expressed during SMX-TM exposure might antagonize *csgD*. Regulators in the horizontally transferred-regions (HTR) associated with control of LEE, including *grlA*, *ler*, and the *pch* genes, were among the transcription factors most strongly affected by SMX-TM exposure. In STEC, five different *pch* genes (*perC* homologs) are encoded within various prophage (Sp) or prophage-like regions (SpLE) (Porter et al., 2005). The *pch* genes can be assigned into three groups based on DNA similarity, size, and functionality: group1 (*pchA*, *pchB*, *pchC*), group 2 (*pchD*), and group 3 (*pchE*). The group 1 *pch*, but not groups 2 and 3, were found to be essential for the full expression of *ler*, *espA*, and attachment of O157:H7 to cultured HEp-2 cells (Iyoda and Watanabe, 2004).

In this study we tested several transcription factors encoded in HTR that were induced during SMX-TM treatment of O157:H7 clinical isolate PA20 (Uhlich et al., 2018) for an effect on *csgD*, *csgD*-dependent cell adhesion, and biofilm properties.

## Materials and Methods

### Strains, Growth Conditions, and Molecular Biology Techniques

All strains used in the study are listed in Supplementary Table S1. *E. coli* serotype O157:H7 PA20 is a clinical isolate that produces Stx1 and Stx2, and carries a prophage insertion in *mlrA* (Uhlich et al., 2013). Strain 20R2R was created by the spontaneous loss of the PA20 Stx1 prophage that restored *mlrA* function and RpoS-dependent *csgD* expression (Uhlich et al., 2016). NEB 5-alpha (New England Biolabs Inc.) and One Shot PIR1 (Invitrogen) were used as *E. coli* host strains for intermediary cloning steps. Strains were cultured in lysogeny broth (LB) prepared using the Miller formulation or LB without added NaCl (LB-NS).

Chromosomal DNA was isolated using the DNeasy Blood and Tissue Kit (Qiagen) or Qiagen Genomic-tip 100/G (Qiagen). The Qiagen multiplex PCR kit was used for routine PCR amplifications (Qiagen). Primers utilized in this research are listed in Supplementary Table S2.

### Mutant Strain Construction

Deletions of the *ler*, *glrA*, *pchA*, *pchB*, *pchC*, *pchD*, *pchE* genes in the indicated strains were made as previously described replacing major portions of the coding regions with neomycin or chloramphenicol cassettes (Uhlich et al., 2009; primers shown in Supplementary Table S2). The cassettes were removed in strains bearing *ler*, *pchC*, and *pchE* deletions to avoid polar effects on downstream genes, except for the neomycin cassette in strain PA20Δ*ler* (GeneBridges GmbH). All deletions were verified by PCR using primers (not listed) designed from areas flanking the homologous recombination regions.

The open-reading-frames, along with sufficient immediate 5′ sequence to include the native ribosomal binding sites of *glrA*, *pchA*, *pchB*, *pchC*, *pchD*, and *pchE* were cloned into plasmid pSE380 (primers in Supplementary Table S2). During testing, strong IPTG induction of certain regulators inhibited host strain growth. Therefore, only un-induced results are shown, taking advantage of the leaky nature of the pSE380 *trc* promoter to generate cloned insert expression sufficient to observe phenotypic differences.

### CR Dye Affinity and Biofilm Assays

Curli expression was tested by monitoring strain CR affinity. Three microliters of LB overnight cultures were spotted on T-medium agar containing 20 mg/l CR dye and 10 mg/l Coomassie brilliant blue G (TA) as described (Collinson et al., 1991; Chen et al., 2013). Plates were incubated for the designated times, imaged using Epson Perfection 3200 Photo Scanner/Epson Scan software (Professional mode) with a light blue background at 600 dots per inch, and compared visually for differences in red color intensity. Biofilm formation was assayed in 96-well format stained with crystal violet (CV) dye, slightly modifying the method described by Chen et al. (2013): plates were covered with sterile adhesive porous rayon films (VWR, Avantor) instead of plastic lids to allow equal gas exchange. Plates were incubated at 30°C for 48 h in humidity chambers and biofilms were heat-fixed at 60°C for 30 min after removing non-adhered bacteria by washing with water.

### RNA Isolation, cDNA Preparation, and qRT-PCR

In order to study the effect of over-expression of HTR regulators on genes involved in curli and biofilm formation (*csgD*, *csgA*), a relative quantification of gene expression was carried out on PA20 and 20R2R strains transformed with pSE380 plasmids carrying different HTR regulatory genes. One milliliter of LB overnight culture of each strain was centrifuged at 7,000 × *g* for 5 min, re-suspended in 100 μl LB, spin-plated on 30°C-pre-warmed T-medium agar containing ampicillin (100 μg/ml), and incubated for 8 h at 37°C or 12 h at 30°C. Every strain was plated in triplicate. Bacteria were harvested with a sterile cotton swab (Puritan Medical Products Company, LLC) and suspended in RNAzol RT (Molecular Research Center). Extraction of total RNA was performed following the manufacturer’s instruction (including phase separation with 4-bromoanisole) and re-suspended in 40 μl nuclease-free water. TURBO DNA-free kit (Ambion) was used to eliminate contaminating DNA from isolated RNA samples. The High Capacity cDNA Reverse Transcriptase Kit (Thermo Fisher Scientific) was used to make cDNA from 1 μg total RNA.

In order to compare *pchE* expression in PA20 grown on agar surfaces at 30°C and 37°C, with and without exposure to 27× SMX-TM, total RNA was isolated and cDNA was made as previously reported (Uhlich et al., 2018). Samples were tested in triplicate.

qRT-PCR was performed by adding 1 μl (20 ng) of cDNA or DNase-treated RNA (negative control) to 19 μl reaction mixture consisting of 0.5 μM of each primer (Integrated DNA Technologies) and 10 μl Fast SYBR Green Master Mix (Applied Biosystems). Primers are listed in Supplementary Table S2. Amplification was carried out on a 7500 Fast Real Time PCR System (Applied Biosystems) using the following parameters: denaturation at 95°C for 20 s followed by 40 cycles (3 s at 95°C, 30 s at 60°C). Melt curve analysis was used to verify the specificity of the amplification products. The *gyrA* gene was used as a reference to normalize the results and PA20+pSE380 or 20R2R+pSE380 were used as calibrator strains. qRT-PCR data were analyzed using the fold change (FC) = 2^−ΔΔCT^ method (Livak and Schmittgen, 2001). The mean FC for three trials of qRT-PCR for the selected genes along with standard deviations (SD) were reported.

### Bacterial Adhesion/Invasion Assay on HEp-2 Cells

The influence of the HTR regulators on bacterial attachment to eukaryotic cells was tested using a HEp-2 cell line (ATCC CCL-23) grown in EMEM, supplemented with 10% fetal bovine serum (all from ATCC), at 37°C in a humidified 5% CO_2_ atmosphere. For adhesion assays, 500 μl of 1 × 10^5^ cells/ml were seeded in a tissue culture treated polystyrene 24-well plates (Sigma-Aldrich) and cultivated until an 85–95% confluent monolayer was formed.

Bacterial strains were grown standing in LB broth with or without ampicillin (100 μg/ml) overnight at 37°C or on T-medium agar with or without ampicillin (100 μg/ml) for 2 days at 30°C. Bacteria were centrifuged and re-suspended (broth cultures) or directly harvested (agar plates) in phosphate buffer saline (PBS). Dilutions in EMEM, with or without ampicillin (100 μg/ml), corresponding to an optical density (OD_600_) of ∼0.07 were prepared for eukaryotic cell exposure. The EMEM covering HEp-2 monolayers was replaced with 1 ml/well of EMEM bacterial suspension and the cells were incubated at 37°C with 5% CO_2_ for 3 h. Afterward, cells were washed five times with PBS to remove unbound bacteria and infected monolayers were treated with 1 ml of 0.1% Triton X-100 for 30 min.

The numbers of cell-associated bacteria was detected by plate count assay. Serial 10-fold dilutions of lysed cells were prepared in PBS and five 10 μl drops of each dilution were placed onto LB agar plates. CFU count was performed after incubation at 37°C for 24 h. The wash PBS was saved and used for CFU counts of unattached bacteria. The percentage of attached bacteria was calculated as follows: n. adhered bacteria × 100/n. adhered bacteria + n. unbound bacteria.

### Curli Extraction, Gel Separation, and Measurement

*Escherichia coli* strains were grown on T-medium agar for 24 h at 37°C. Cells were collected from individual plates and analyzed for total soluble proteins as described previously (Uhlich et al., 2009) with modifications. It is necessary to treat polymerized curli with formic acid (pH 2.0) before gel separation (Collinson et al., 1991). Additionally, it is also our experience that the treated samples must be maintained on ice for all steps of the extraction and gel sample preparation process. Cells were collected from plates into sterile water and the optical densities for all strains were adjusted to 0.5 (600 nm). Additionally, aliquots were taken from each individual strain sample and plated onto LB agar to ensure that equal numbers of cells were present in each preparation (data not shown). One milliliter of cells were pelleted for each strain by centrifugation and resuspended in 100 μl of 98% formic acid and incubated for 30 min on ice. After incubation the formic acid solutions were evaporated under a stream of nitrogen while the tubes remained on ice. Dried samples were resuspended in 20 μl LDS buffer (Thermo Fisher Scientific), plus 8 μl 50 mM 1,4-dithiothreitol (DTT) solution and 52 μl water. Samples were incubated for 10 min on ice and 20 μl of each sample were loaded directly onto the gel without exposure to heat. Samples were separated on a 12% Nu-PAGE Bis-Tris gel (Thermo Fisher Scientific) as described previously (Laemmli, 1970) and then stained with Bio-Safe Coomassie stain (Bio-Rad Laboratories, Hercules, CA, United States). The gel was visualized using a Gel Doc^TM^ EZ Imager (Bio-Rad Laboratories) and the relative amounts of the gel bands, for each strain, containing the major curli subunit CsgA were determined using the densitometry function of the Image Lab^TM^ (version 6.0) program (Bio-Rad Laboratories).

### Mass Spectroscopy and CsgA Major Peptide Quantification

The protein bands for each strain containing CsgA and previously measured by densitometry were excised from the gel with sterile scalpels. The gel bands were destained with a methanol solution and the proteins extracted from the gel using 50% acetonitrile, 5% trifluoroacetic acid (TFA) solution. The extracted proteins were digested overnight using Trypsin Gold (Promega, Madison, WI, United States) following the manufacturers’ protocol. Samples were treated with 2% TFA to stop trypsin digestion. Each of the resulting three digested gel extracts were analyzed in triplicate. This process used a Nano-Acquity UHPLC (Waters Co., Milford, MA, United States) running in the trap mode and equipped with a 20 mm × 180 μm, 5 μm Symetry C18 trap column (Waters) and a 200 mm × 75 μm, 1.8 μm HSS T3 (Waters) analytical column using a gradient of water:acetonitrile (0.1% formic acid) running with the composition from 95:5 to 50:50 with a duration of 30 min and a flow rate of 450 nl/min. The nano-UHPLC flow was directed to a Q-Exactive Plus orbitrap mass spectrometer (Thermo Fisher Scientific, Madison, WI, United States) using a nanospray Flex Ion source (Thermo Fisher Scientific). The mass spectrometer data was analyzed with Proteome Discover 2.2 (Thermo Fisher Scientific) using SEQUEST as the database search algorithm and the *E. coli* protein database from UniProt. In order to compare the concentration of the identified protein (CsgA, P28307) the triple charged most abundant peptide with the sequence NSDLTITQHGGGNGADVGQGSDDSSIDLTQR was selected for area integration on the total ion current (TIC) chromatogram. Sample variation was controlled for with protein P11457, expressed at similar levels in each of the three samples. Additionally, prior to HPLC separation and mass spectrometer injection samples were spiked with equal concentrations of rabbit glycogen phosphorylase protein (P00489) for normalizing one MS run to the next.

### Statistical Analyses

For biofilms, the mean optical density values of eluted CV at 590 nm (OD590) for each sample were analyzed as follows. For large screening assays testing the effects of multiple HTR regulators, samples were analyzed in R (R Core Team, 2017) as previously described (Uhlich et al., 2018). Strain variance was assessed with the Bartlett’s test and OD value differences were determined using the non-parametric Kruskal–Wallis test. Multiple pairwise *t*-test comparisons were performed to determine the dye retention differences between grouped strains. The *t*-tests were performed without the assumption of equal variance (pairwise.t.test option pool.SD = F) and the resulting *P*-values were adjusted with the Benjamini-Hochberg procedure. For comparison of HTR deletions in 20R2R and for the focused studies comparing over-expression of *pchA* and *pchE*, data were analyzed using an analysis of variance (ANOVA) for a randomized complete block design. The blocks were identified as the independent replicates. The appropriate term for testing for treatment effects and separating treatment means is the mean square for treatment by block, ignoring the within block well-to-well variation. Treatment means were separated using a Bonferroni least significant difference (LSD) method with *P* < 0.05.

For adhesion/invasion assays, strain samples were tested in triplicate to establish challenge-dose culturing parameters and in quadruplicate for the assays testing the effects of PchA and PchE. For the challenge-dose culturing parameters assay, data were analyzed in R (R Core Team, 2017) as described for the large screening biofilm assays testing the effects of multiple HTR. For the assays testing the effects of PchA and PchE, data were transformed by the square root transformation to correct for non-additivity. The transformed data were analyzed by anova using well-to-well variation as an error term to test for treatment effects and for treatment means separation. The Bonferroni LSD method was used to separate the treatment means (*P* < 0.05). The inferences based on the transformed values were then applied to the untransformed treatment means. As above, any two treatment means with no letter in common are significantly (*P* < 0.05) different by the Bonferroni LSD method.

## Results

Clinical isolate PA20 (*stx*_1_^+^, *stx*_2_^+^) carries a prophage insertion in *mlrA* that restricts *csgD* expression, eliminates biofilm formation, and reduces curli production and CR dye affinity (Uhlich et al., 2013). A spontaneous excision of the PA20 Stx1 prophage reformed the *mlrA* gene and created strain 20R2R (*stx*_1_^−^, s*tx*_2_^+^), which has restored *csgD* expression, full CR affinity, and produces strong biofilms (Uhlich et al., 2016). Strain 20R2R is used in this study to show the effects of the tested regulators on an O157:H7 strain that maintains temperature-dependent (≤30°C) *csgD* regulation and strong curli expression driven from the typical *mlrA*/*rpoS* regulatory network; a strain type isolated more frequently from non-clinical isolates (Biscola et al., 2011; Uhlich et al., 2013).

### Plasmid Induced *pchE* Represses Curli-Dependent CR Affinity in PA20 and 20R2R

In a previous study, certain SMX-TM concentrations decreased PA20 *csgD* expression at 30°C but strongly induced genes in HTR, including virulence regulators *ler*, *grlA*, and *pch* group 1 genes (Uhlich et al., 2018). To determine whether HTR-encoded LEE regulators control *csgD*, we expressed pSE380-cloned *grlA*, *pchA*, *pchB*, *pchC*, *pchD*, or *pchE* in wild-type and mutant strains of PA20 and 20R2R, and tested for differences in *csgD*-dependent phenotypes.

**FIGURE 1:**
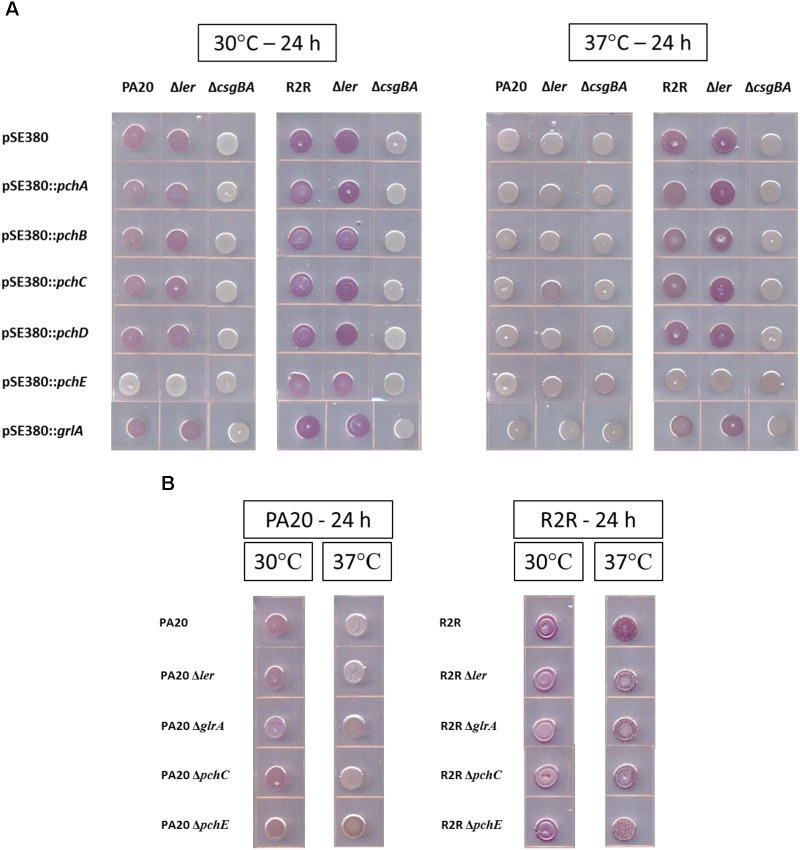
Comparison of the effects of over-expression **(A)** or deletion **(B)** of different horizontally transferred-regions (HTR) on the Congo red dye affinity of strains PA20 and 20R2R. **(A)** Wild-type, Δ*ler*, and Δ*csgBA* strains of PA20 and 20R2R, transformed with control plasmid pSE380 or pSE380 carrying cloned HTR regulator genes, spotted on TA and incubated at 30 or 37°C for 24 h. **(B)** PA20, 20R2R, and strains of each with deletion of *ler*, *grlA*, *pchC*, and *pchE* genes spotted on TA and incubated at 30 and 37°C for 24 h.

At 30°C, PA20 pSE380 showed a light red color on TA (Figure 1A). Deletion of *csgBA* (PA20Δ*csgBA* pSE380) resulted in loss of all color but deletion of *ler* (PA20Δ*ler* pSE380) elicited little change in CR affinity. Expression of *pchE* eliminated all red color in PA20 and PA20Δ*ler* indicating a strong *pchE* repressive effect on curli that is independent of *ler*. Plasmid expression of *pchA*, *pchB*, and *pchC* caused a slight reduction in PA20 red color but *pchD* and *grlA* did not change the curli-dependent dye affinity. In PA20Δ*ler*, none of the cloned regulators, other than *pchE*, affected the dye affinity indicating that the *pchA*, *pchB*, and *pchC* reductions were *ler*-dependent. At 37°C, a non-permissive temperature for *rpoS*-dependent curli expression, dye affinity was minimal in PA20 pSE380 and the mutants, whether carrying cloned regulators or the control plasmid. Only *pchC* expression produced a small color increase in PA20Δ*ler* that was more brown than red.

The curli-producing strain, 20R2R, and its mutant derivative, 20R2RΔ*ler*, displayed strong red colony staining when carrying pSE380 at both 30 and 37°C (Figure 1A). All red color was lost in 20R2RΔ*csgBA* carrying pSE380 at either temperature confirming the complete dependence of CR affinity on curli. At 37°C, plasmid expression of *pchE* eliminated all CR binding in strains 20R2R and 20R2RΔ*ler*. At 30°C, the optimum temperature for RpoS-dependent gene expression, 20R2R and its *ler* mutant would be expected to show their strongest CR affinity. Under such conditions, *pchE* expression reduced but did not eliminate CR staining. All other tested regulators had little effect on CR affinity at either 30 or 37°C. Therefore, *pchE* was the only tested HTR regulator that strongly affected CR binding in strains PA20 and 20R2R, repressing dye affinity at both temperatures in a *ler*-independent manner.

### Basal *pchE* Expression Represses PA20 CR Affinity

The expression level of certain HTR regulators, such as the group 1 *pch*, from plasmid pSE380 would be similar to that expected following strong induction of the SOS response (Uhlich et al., 2018). However, HTR-encoded regulators could also influence *csgD*-dependent properties during non-inducing conditions. To test for such effects, CR affinity differences between parent strains and strains bearing deletions of *ler*, *grlA*, *pchE*, or group 1 *pch* representative, *pchC*, were determined under non-inducing conditions (Figure 1B). Loss of each regulator had minimal effect of PA20 CR affinity except for *pchE* whose deletion resulted in slightly increased red colony color at 37°C, indicating that basal expression of *pchE* represses curli formation at host temperatures (additional images of the effect of *pchE* deletion on CR affinity are shown in Supplementary Figure S1). Deletion of regulator *grlA* also resulted in a color change compared to the parent but it was weaker in intensity and more brown than that induced by *pchE* deletion. In 20R2R, deletion of individual regulators caused no visible color changes at either 30 or 37°C. It is not clear whether *pchE* failed to repress curli production in 20R2R or whether *pchE*-induced reductions were too subtle to be visible in the stronger curli-producing strain. Therefore, *pchE* was the only *pch* homolog controlling curli production under conditions where prophage elements were not induced by DNA damage, maintaining a slight repression in strain PA20.

### *PchE* Is a Strong Repressor of Biofilm at 30°C

**FIGURE 2:**
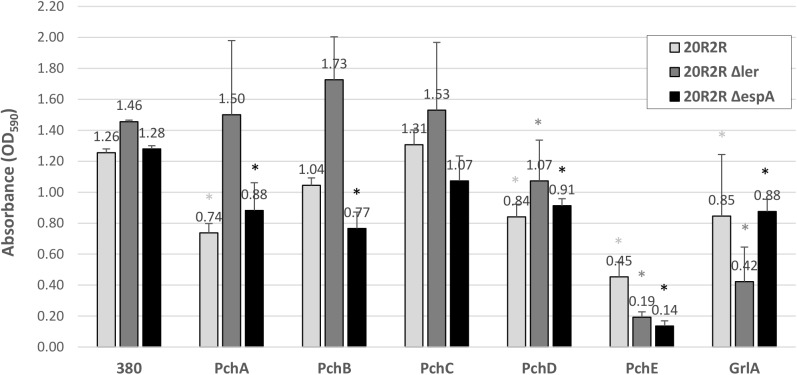
Comparison of the effect of different HTR regulators on *E. coli* O157:H7 strain 20R2R biofilm formation on polystyrene. Crystal violet dye retention by strains 20R2R, 20R2RΔ*ler*, and 20R2RΔ*espA* transformed with control plasmid pSE380 or pSE380 carrying cloned HTR regulatory genes following 48 h incubation in polystyrene 96-well plates at 30°C in LB-NS. Bars represent the mean CV absorbance measured at 590 nm (OD_590_) ± standard deviation (SD) of two independent samples. Stars indicate statistical differences (*P* ≤ 0.01) between each sample and the relative control (star color indicates the relative control). *P*-values for every sample are reported in Supplementary Table S3.

**FIGURE 3:**
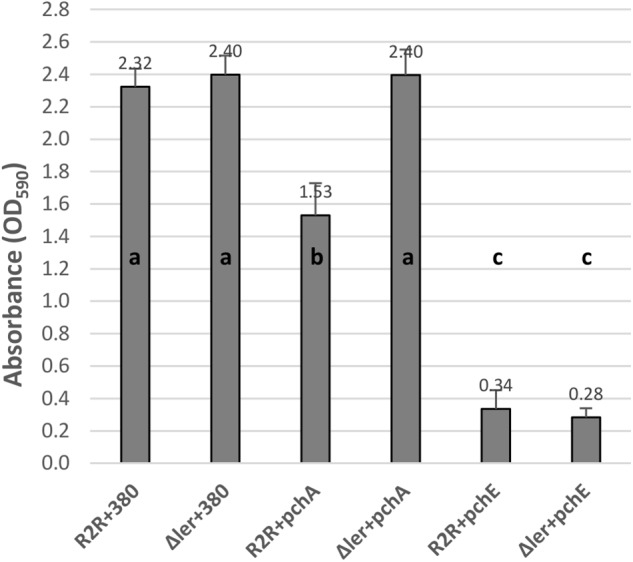
Comparison of the effect of regulators PchA and PchE on *E. coli* O157:H7 strain 20R2R biofilm formation on polystyrene. Crystal violet dye retention by strains 20R2R and 20R2RΔ*ler* transformed with control plasmid pSE380 or pSE380 carrying cloned *pchA* or *pchE* following 48 h incubation in polystyrene 96-well plates at 30°C in LB-NS. Bars represent the mean CV absorbance measured at 590 nm (OD_590_) ± SD of four independent samples. Any two means with no letter in common are significantly (*P* < 0.05) different by the Bonferroni LSD method.

The effects of HTR regulators on biofilm formation at 30°C and 37°C were tested by expressing the cloned regulators in strains PA20, 20R2R, and mutant strains of each with deletion of *ler* and *espA*. 20R2R pSE380 did not produce biofilm at 37°C and PA20 pSE380 failed to form biofilm at either temperature (data not shown). Over-expression of the HTR regulators and deletions of *ler* and *espA* did not alter CV binding appreciably under those same strain/temperature conditions (data not shown). Therefore, only the results of strains 20R2R, 20R2RΔ*ler*, and 20R2RΔ*espA* carrying the control plasmid or cloned regulators at 30°C are shown (Figure 2). To test multiple regulators in several different strain backgrounds under identical conditions we screened two independent samples per strain in a 96-well format and report mean CV dye absorbance values (Figure 2). A statistical analysis of every possible strain pair comparison is shown in the supplemental information (Supplementary Table S3). Deletion of *ler* in 20R2R slightly increased mean CV retention (*P* > 0.05) but *espA* deletion did not affect biofilm formation. Expression of the group 1 *pch* genes, except for *pchC*, lowered mean CV retention of both the wild-type 20R2R and the EspA mutant but not of the *ler*-deletion strain suggesting that strong group 1 gene induction causes modest biofilm reductions (<50%) by a *ler*-dependent, but *espA* independent, mechanism. In contrast, group 3 regulator, *pchE*, strongly reduced CV retention (>3-fold) in 20R2R, 20R2RΔ*ler*, and 20R2RΔ*espA* (*P* < 0.05) indicating an *espA*- and *ler*-independent mechanism. Regulators GrlA and PchD also slightly reduced the mean CV binding in 20R2R and the mutants, but variation between replicates made interpretation more difficult. A second study with additional independent samples was conducted to confirm the observed trends focusing on the group 1 and group 3 *pch* regulators and the suppressive mechanisms associated with *ler*. Regulator *pchA* was tested alone as a representative of group 1 *pch*. As shown in Figure 3, no significant difference in CV binding was observed between 20R2R and 20R2RΔ*ler* (*P* > 0.05) but plasmid-based *pchA* expression reduced (*P* < 0.05) 20R2R CV binding. The reduction was not evident when *pchA* was plasmid expressed in 20R2RΔ*ler*, confirming a *ler*-dependent suppressive action for PchA. Over-expression of *pchE* reduced biofilm formation nearly sevenfold in both 20R2R and 20R2RΔ*ler* confirming the strong, *ler*-independent, *pchE* biofilm suppression.

**FIGURE 4:**
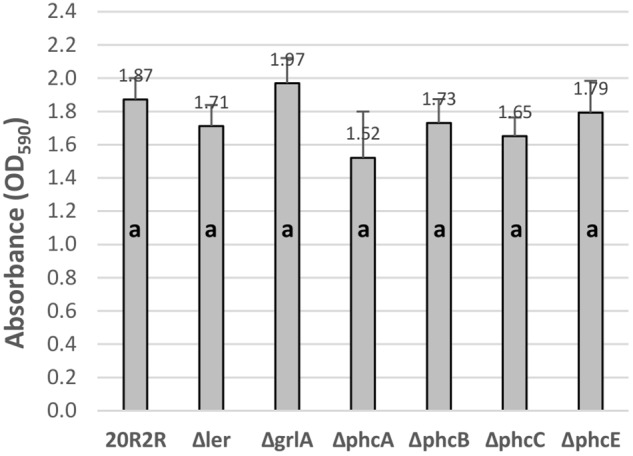
Comparison of the effect of the deletion of different HTR regulators on *E. coli* O157:H7 strain 20R2R biofilm formation on polystyrene. Crystal violet dye retention by strains 20R2R and 20R2R carrying deletions of certain HTR regulators following 48 h incubation in polystyrene 96-well plates at 30°C in LB-NS. Bars represent the mean CV absorbance measured at 590 nm (OD_590_) ± SD of two independent samples. Any two means with no letter in common are significantly (*P* < 0.05) different by the Bonferroni LSD method.

To test for regulatory effects by the various HTR regulators under conditions where the prophage elements were not induced, we compared biofilm formation between wild-type 20R2R and strains with deletions of the individual regulators at 30°C (Figure 4). We tested a deletion of *ler* rather than *pchD* in our 8-strain test protocol. There were no significant differences attributed to deletion of any regulator compared to controls indicating that the tested regulators have little effect on biofilm formation under non-SOS-inducing, environmental growth conditions.

### Prophage Induction Reduces *pchE* Expression at 37 and 30°C

The strong CR affinity and biofilm effects of *pchE* being the result of plasmid expression, it was deemed necessary to confirm transcription and regulation of *pchE* when it is encoded on the chromosome. In the previous RNA-Seq study of PA20 (Uhlich et al., 2018), *pchE* transcripts were identified in broth samples grown at 30°C and SMX-TM induction reduced their expression nearly twofold. We extended the *pchE* expression studies to include host temperatures using qRT-PCR, comparing PA20 grown on agar surfaces at 30 and 37°C, with and without exposure to SMX-TM. At 30°C, there was a greater than sixfold (*SD* = 1.5) decrease in *pchE* expression in SMX-TM-treated samples compared to the un-treated controls. When tested at 37°C, *pchE* expression was reduced more than sevenfold (*SD* = 0.4) by SMX-TM exposure. Therefore, *pchE* is expressed in PA20 at both host and environmental temperatures and prophage-inducing antibiotics reduce its expression at both temperatures (data not shown).

### PchE Represses *csgD* and *csgBA* Expression

**FIGURE 5:**
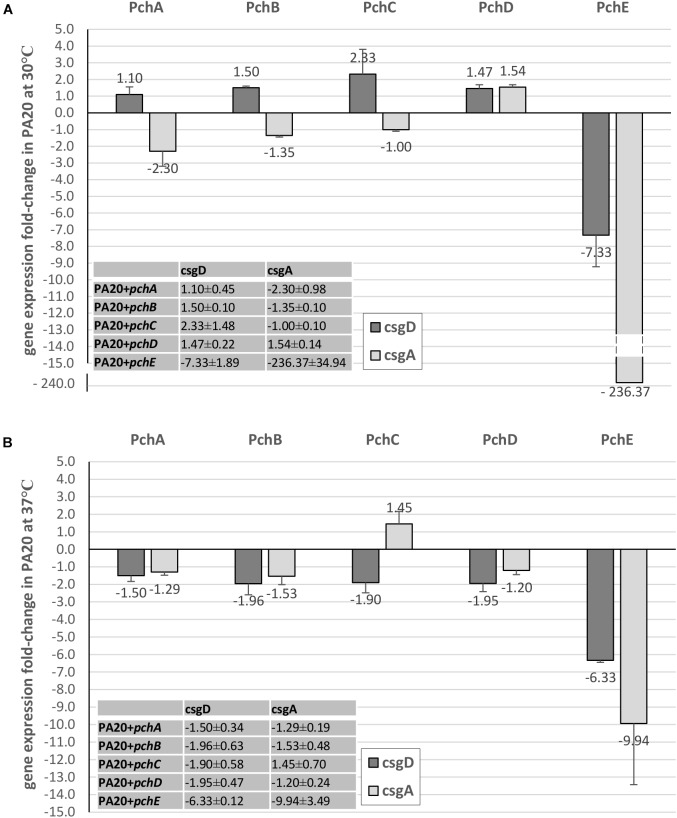
Comparison of the effect of Pch regulators on *csgD* and *csgA* expression in strain PA20. Fold change (FC) *csgD* and *csgA* expression differences between PA20 carrying control plasmid pSE380 and PA20 carrying pSE380 with cloned *pch* homologs at 30°C **(A)** and 37°C **(B)**. qRT-PCR was performed on cDNA derived from three independent samples of strains PA20 or 20R2R grown on T-medium agar at 30 or 37°C and analyzed using the 2^–ΔΔCT^ method. Bars represent the mean FC of the three independent samples along with SD.

**FIGURE 6:**
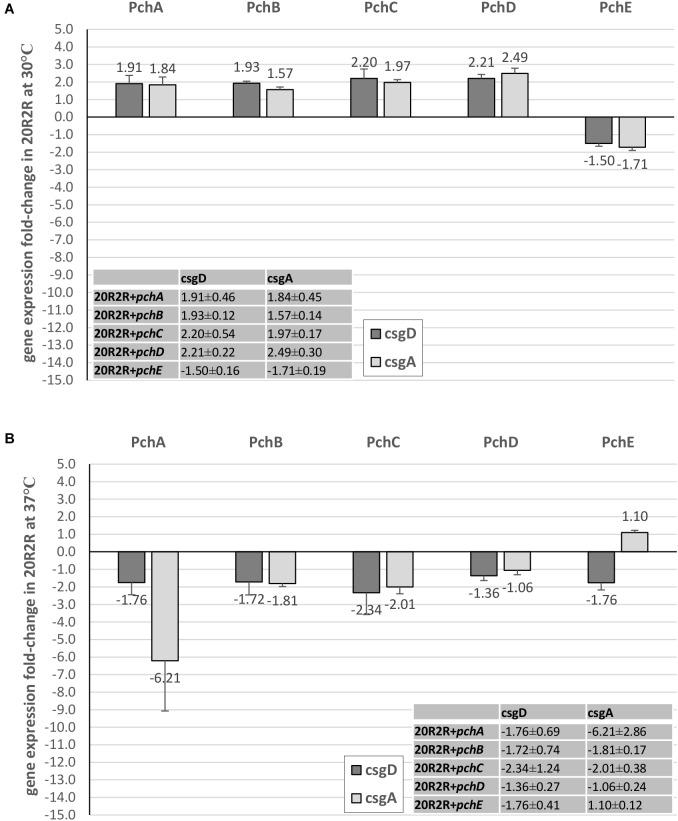
Comparison of the effect of Pch regulators on *csgD* and *csgA* expression in strain 20R2R. Fold change (FC) *csgD* and *csgA* expression differences between 20R2R carrying control plasmid pSE380 and 20R2R carrying pSE380 with cloned *pch* homologs at 30°C **(A)** and 37°C **(B)**. qRT-PCR was performed on cDNA derived from three independent samples of PA20 or 20R2R strains grown on T-medium agar and analyzed using the 2^−ΔΔCT^ method. Bars represent the mean FC of the three independent samples along with SD.

A likely control point for the regulation of curli-dependent functions by the HTR regulatory genes would be the *csgBA* structural genes or their master regulator, *csgD*. Expression of *csgD* and *csgA* were compared between strains carrying plasmid expressed *pch* regulators and the control plasmid (Figures 5, 6). At 30°C, *pchE* decreased PA20 *csgD* expression >7-fold and *csgA* expression >280-fold compared to the control, findings that correlate well with the complete loss of CR affinity in PA20 under those conditions (Figure 5A). At 37°C, *pchE* decreased PA20 *csgD* expression ≥6-fold and *csgA* expression nearly 10-fold (Figure 5B). The five other *pch* genes caused only small changes in *csgD* and *csgA* expression at either temperature (Figure 5).

In strain 20R2R at 30°C, both *csgD* and *csgA* expression were increased by the *pchA*, *pchB*, *pchC*, and *pchD* but decreased by *pchE*. However, all changes were <2-fold compared to the control strain (Figure 6A). At 37°C, where plasmid-based expression of *pchE* strongly reduced CR affinity, each of the five *pch* resulted in only slight *csgD* repression (<2-fold). *csgA* was also affected little by the *pch* genes at 37°C. Only *pchA* induced a *csgA* expression decrease greater than twofold and there was substantial replicate variability in that comparison (Figure 6B). Therefore, while *pchE* strongly repressed PA20 *csgD* and *csgA* transcripts at both host and environmental temperatures, a similar response did not accompany the marked reduction of CR binding in 20R2R at 37°C. While it is possible that *pchE* controls 20R2R CR affinity through a different pathway than in PA20, a more likely explanation is that the time-point at which 20R2R transcripts were harvested was not appropriate to capture the expression changes.

### Induced *pchE* Represses CsgA

**FIGURE 7:**
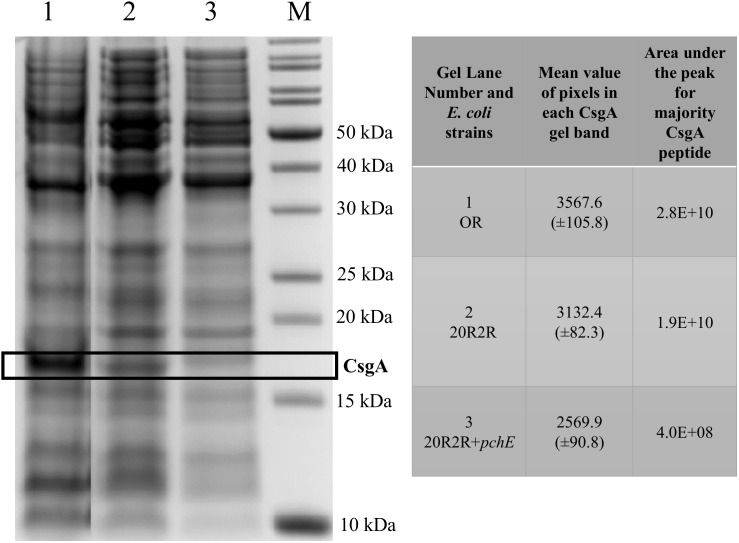
A 12% Bis-Tris polyacrylamide gel containing formic acid treated whole cell extracts and the resulting data derived from the CsgA containing band. *E. coli* strains grown on T-medium agar at 37°C; OR (lane 1), 20R2R (lane 2), 20R2R+*pchE* (lane 3), molecular weight standards (lane M). Box marks the location of the bands containing the CsgA protein. The mean value of pixels present in the individual CsgA bands were visualized and measured by a Gel Doc^TM^ EZ Imager and Image Lab^TM^ (Bio-Rad) program. The area under the peak for the major peptide (NSDLTITQHGGGNGADVGQGSDDSSIDLTQR [triple charged]) produced from tryptic digestion of the CsgA containing band were measured by mass spectrometry.

To confirm that curli expression was responsible for the CR affinity differences in strain 20R2R following expression of *pchE* at 37°C we compared the concentrations of the major subunit of curli, CsgA, produced by 20R2R containing either pSE380 or pSE380::*pchE*, each isolated from equal numbers of cells. The SDS–PAGE separation of total formic acid soluble proteins is shown in Figure 7. Strain 43894OR, known to greatly overexpress curli, was included in this assay to help localize CsgA on the gel (Uhlich et al., 2001). Strain 43894OR and 20R2R each produced a protein band that migrated to a position corresponding to approximately 16 kDa. This size band was significantly decreased in appearance when strain 20R2R carried pSE380::*pchE*. The relative amounts of the CsgA containing bands produced by the different strains were quantitated by using densitometry to measure the mean value of pixels present in the CsgA containing band in the gel image (Figure 7). The CsgA containing band of 20R2R was determined to contain a significantly greater pixel amount compared to 20R2R+pSE380::*pchE* suggesting that the band possessed a greater concentration of that protein (Figure 7). To confirm this, the CsgA containing bands previously measured by densitometry were excised, trypsin digested and the resulting majority CsgA peptide identified and quantitated by measuring the resulting area under the peak of the peptide by using UHPLC separation and mass spectrometry. The majority CsgA peptide was specifically determined to be present in significantly greater amounts in the proteins derived from the gel band specific to 20R2R compared to those of 20R2R pSE380::*pchE*, as shown in the table in Figure 7.

### *pchE* Over-Expression Represses Curli-Dependent HEp-2 Cell Adhesion

**FIGURE 8:**
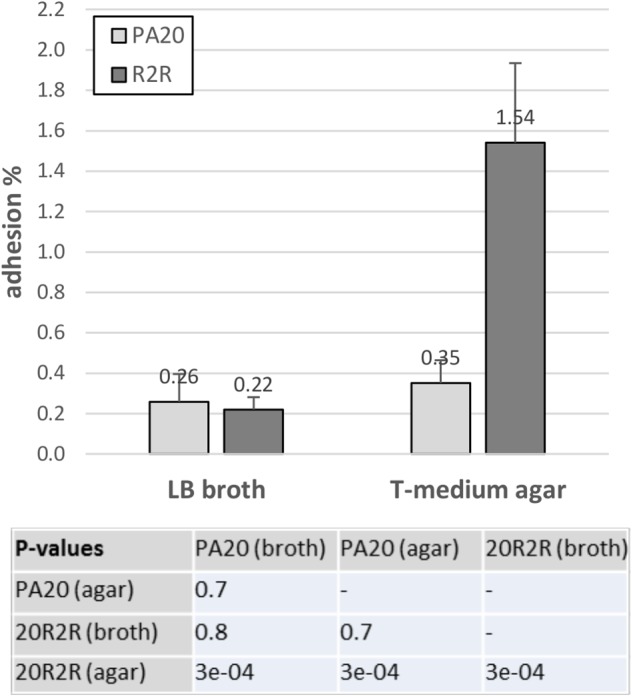
Influence of different growth conditions on the adhesion of strain PA20 and 20R2R to HEp-2 cells. Percentage of adhered PA20 and 20R2R to HEp-2 cells after 3 h exposure at 37°C in 5% CO_2_. Strains were grown in LB broth or on T-medium agar prior to challenge. Bars represent the mean adhesion percentage ± SD of three independent samples.

**FIGURE 9:**
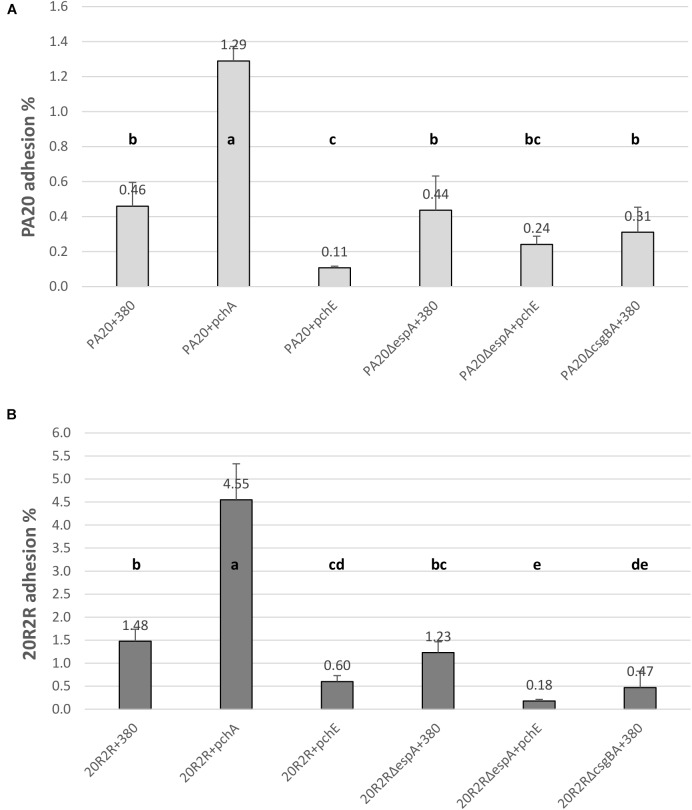
Comparison of the effect of HTR regulators PchA and PchE on the adhesion of *E. coli* O157:H7 strain PA20 and 20R2R to HEp-2 cells. Percentage of adhered wild-type, Δ*espA*, and Δ*csgBA* PA20 **(A)** and 20R2R **(B)** transformed with control plasmid pSE380 or pSE380 carrying *pchA* or *pchE* regulators to HEp-2 cells following 3 h exposure at 37°C in 5% CO_2_. Bars represent the mean adhesion percentage ± SD of four independent samples. Any two treatment means with no letter in common are significantly (*P* < 0.05) different by the Bonferroni LSD method.

The HTR regulators had strong effects on curli-dependent CR affinity on TA, especially in strain 20R2R at 37°C. However, biotic surfaces would be a more relevant target for adhesive curli fibers at 37°C. Both curli and EspA are involved in adhesion to eukaryotic tissue; EspA playing an important role in the intimate cell attachment required for LEE TTSS secretion and curli by more general attachment to a variety of human serum and tissue proteins (Olsén et al., 1993, 1998; Sjöbring et al., 1994; Ben Nasr et al., 1996). Therefore, we also tested the effects of certain HTR regulators on HEp-2 cell adhesion using mutant and wild-type strains of PA20 and 20R2R. Because growth conditions determine curli expression, cell adhesion of parental strains was first compared using two different methods for preparing the challenge dose: growth in LB broth for 18 h at 37°C and growth on T-medium agar for 48 h at 30°C (Figure 8). HEp-2 cell adherence (conducted at 37°C) did not differ (*P* > 0.05) between PA20 strains cultured by the two different methods but the percentage of adhered 20R2R was more than fourfold greater (*P* < 0.05) when the bacteria were collected from agar rather than from broth. Therefore, we harvested all challenge strains from T-medium agar after 48 h at 30°C. We focused the adhesion studies on the group 1 *pch* and *pchE*, the most active regulators in CR-binding and biofilm assays, and used *pchA* as the representative for the group 1 genes (Figure 9). We also confirmed the repressive effect of *pchE* on *csgD* at 37°C in EMEM using qRT-PCR. Plasmid-based expression of *pchE* reduced PA20 *csgD* expression threefold (results not shown).

Elimination of curli fimbriae by *csgBA* deletion resulted in significant reductions in 20R2R cell adhesion, confirming the curli role in cell adhesion (Figure 9B). In PA20, where curli formation is already restricted by *mlrA* disruption, *csgBA* deletion reduced the mean adhesion percentage but it did not meet significance (*P* > 0.05) (Figure 9A). When *pchE* was over-expressed, the adhesion percentages of both PA20 and 20R2R were reduced greater than twofold (*P* < 0.05) compared to the plasmid only controls. When *espA* was deleted in either PA20 pSE380 or 20R2R pSE380, there was no significant reduction in cell adhesion (*P* > 0.05) compared to the parent control strains. As group 1 *pch* are proven enhancers of *espA*, it is likely that they were not induced under the conditions of this study. However, when *pchA* was expressed from plasmid pSE380, HEp-2 cell adhesion was indeed increased nearly threefold in both PA20 and 20R2R (Figure 9). Finally, in the weak curli-producing strain PA20, but not in 20R2R, high *pchE* expression reduced cell adhesion to levels lower than those detected following *csgBA* deletion, which suggests that *pchE* may control factors other than curli that are involved in cell adhesion. Collectively, the cell studies indicate that the regulation of O157:H7 adhesion to host tissue requires the coordinated expression of various *pch* homologs acting on different protein adhesion factors.

## Discussion

A high proportion of O157:H7 clinical strains carry the Stx1 prophage inserted in the gene encoding MlrA, a transcription factor required for RpoS-dependent expression of the *E. coli* central biofilm regulator, *csgD* (Uhlich et al., 2013). Stress resistance properties in clinical strains could be enhanced by prophage excision or run-off *mlrA* transcription that would restore *csgD* function (Uhlich et al., 2016). However, a past study in strain PA20 showed that SOS induction repressed rather than activated *CsgD*, while simultaneously activating horizontally transferred prophage and prophage-like elements, including LEE (Uhlich et al., 2018). In the present study, certain HTR regulators were over-expressed from a plasmid to define their role in the observed *csgD* repression and to test their effect on O157:H7 adhesive properties during environmental and host conditions. In addition to clinical strain PA20, we also tested 20R2R, a derivative of PA20 that had spontaneously excised the Stx2 prophage, reforming *mlrA*, and restoring a biofilm-proficient state more adapted for environmental conditions. Our results have identified *pchE* as a negative regulator of *csgD* expression and CsgD-dependent phenotypes such as curli production, biofilm formation, and adhesion to cultured human epithelial cells. The five EHEC homologs of the EPEC *perC* gene have been studied extensively and a clear role for *pchA*, *pchB*, and *pchC* in the control of LEE through regulation of *ler* has been demonstrated. However, *pchD* and *pchE* had little effect on the LEE operons and their major function(s) have remained elusive (Iyoda and Watanabe, 2004; Porter et al., 2005; Uhlich et al., 2018).

At 30°C, plasmid-expression of *pchE* strongly repressed the expression of both *csgD* and *csgA* in PA20 but our previous RNA-Seq study and the qRT-PCR results herein both indicate that SMX-TM treatment reduces rather than increases *pchE* expression, which would subsequently increase *csgD* expression. Therefore, it is unlikely that PchE controlled the reductions in *csgD* expression reported in our previous SMX-TM challenge study. When expressed individually, the group 1 *pch* genes had minor effects on *csgD*. However, *pchA* and *pchB* both reduced *csgA* expression and biofilm formation, and a combined regulatory effect by the group 1 members, similar to the strategy used for group 1 enhancement of LEE (Porter et al., 2005), could provide significant *csgD* repression. Therefore, members of the group 1 *pch* were more likely to be responsible or partially responsible for the reduced *csgD* expression following SOS induction.

Although it is unlikely that PchE was responsible for the *csgD* repression following SMX-TM exposure, these results indicate that it is a strong *csgD* repressor that functions using a different, Ler-independent, mechanism than the group 1 *pch*. It is not clear what benefits PchE repression of *csg*D would provide for O157:H7 strains during growth at 30°C. PchE-imposed inhibition of curli expression seems counter-productive for environment survival; although, SOS initiation during stress could relieve the *csgD* repression. It is also conceivable that the reduction of *csgD* expression would minimize energy costs and disperse biofilm communities to aid in survival during extreme nutrient deprivation. A full understanding of the role of PchE in environmental survival will require full elucidation of the initiating cues and regulatory control of this strong curli regulator.

When the HTR regulators were tested for regulatory effects on clinical isolate PA20 at 37°C, both the group 1 *pch* homologs and group 3, *pchE*, affected adhesion to cultured HEp-2 cells. The group 1 *pch* genes, following SOS induction, are proven strong enhancers of adhesion to HEp-2 cells through the *ler*-dependent expression of *espA*, but the role of curli in HEp-2 adhesion has been unclear (Iyoda and Watanabe, 2004; Sharma et al., 2016). In this study, a small but consistent increase in CR binding following *pchE* deletion in clinical strain PA20 indicated that PchE repressed *csgD* and curli during growth when the SOS response was not induced. When strongly expressed, group 1 *pchA* generated the highest percentages of adherent PA20 and 20R2R, but under non-inducing conditions, *espA* was dispensable for the attachment of either strain, and the attachment of 20R2R was clearly supported by curli. Therefore, curli likely maintains a low level of non-specific cell attachment under non-induced conditions. Induction of the SOS would likely increase both curli-mediated adhesion, by decreasing *pchE*, and intimate adhesion, by increasing the group 1 *pch*.

The significance of strong curli expression for STEC attachment to host cells and disease pathogenesis was dramatically shown by the serotype 104:H4 strain responsible for a large outbreak of bloody diarrhea with HUS in Germany (Richter et al., 2014). Shiga toxin acquisition by a strain with an enhanced ability to adhere to the gut and initiate inflammation due to unusually high curli production resulted in high death losses and high incidence of HUS. In contrast, typical clinical strains of serotype O157:H7, arguably the most common disease-producing STEC serotype, have more successfully adapted to human hosts through strategies that curtail *csgD* expression (Uhlich et al., 2013). PchE-mediated repression of curli during non-inducing conditions may provide an additional mechanism to increase that host adaptation. Being capable of repressing CsgD expression and cell adhesion in low curli-expressing clinical strains until reversal by SOS-induction to support group 1 *pch* initiation of intimate adhesion adds an additional important layer of fine control to serotype O157:H7 virulence regulation.

In this study, we determined that PchE is a strong *csgD* repressor, defining a function for the group 3 homolog; however, it was clearly not responsible for *csgD* repression during SOS induction. Moreover, its regulatory role at 30°C is unclear, and will requiring additional studies to identify the specific conditions controlling its expression. Under host conditions when the group 1 *pch* and *espA* were not expressed, PchE affected curli-dependent HEp-2 cell adhesion suggesting a role in controlling early host cell attachment. Studies defining the PchE regulon and its regulatory mechanisms will help to define this potentially important O157:H7 regulatory factor.

## Author Contributions

EA, LR, and GU performed the cell culture and adhesion assays. GU, EA, and BC performed the DNA experiments. NG and AN performed the protein experiments. ER and EA carried out statistical analysis. EA, GU, NG, and ER prepared the manuscript. All authors reviewed the manuscript.

## Conflict of Interest Statement

The authors declare that the research was conducted in the absence of any commercial or financial relationships that could be construed as a potential conflict of interest.
