# Supplementary material for: Pch Genes Control Biofilm and Cell Adhesion in a Clinical Serotype O157:H7 Isolate
Source: Front Microbiol. 2018 Nov 23;9:2829. doi: 10.3389/fmicb.2018.02829 (PMC6265319; doi:10.3389/fmicb.2018.02829)

Supplementary Material

Pch genes control biofilm and cell adhesion in a clinical serotype O157:H7 isolate

**Elisa Andreozzi^1$^, Nereus W. Gunther IV^1^, Erin R. Reichenberger^1^, Luca Rotundo^1^, Bryan J. Cottrell^1^, Alberto Nuñez^1^ and Gaylen A. Uhlich^1$*^**

*** Correspondence:** Gaylen Uhlich: gaylen.uhlich@ars.usda.gov

# Supplementary Figures and Tables

**Supplementary Table 1.** Bacterial strains used in this study.

| **Strain** | **Strain name** | **Description** |
| --- | --- | --- |
| PA20 | PA20 | *E. coli* serotype O157:H7 clinical isolate |
| PA20 Δ*ler* | PA20Δ*ler* | PA20 deletion of *ler*, *grlA*, *pchC* or *pchE* genes |
| PA20 Δ*grlA* | PA20Δ*grlA* |  |
| PA20 Δ*pchC* | PA20Δ*pchC* |  |
| PA20 Δ*pchE* | PA20Δ*pchE* |  |
| PA20+pSE380 | PA20+pSE380 | PA20 transformed with pSE380 |
| PA20+pSE380::*pchA* | PA20+*pchA* | PA20 transformed with pSE380 carrying *pchA*, *B*, *C*, *D*, *E* or *grlA* genes |
| PA20+pSE380::*pchB* | PA20+*pchB* |  |
| PA20+pSE380::*pchC* | PA20+*pchC* |  |
| PA20+pSE380::*pchD* | PA20+*pchD* |  |
| PA20+pSE380::*pchE* | PA20+*pchE* |  |
| PA20+pSE380::*grlA* | PA20+*grlA* |  |
| PA20 Δ*ler*+pSE380 | PA20Δ*ler*+pSE380 | PA20 Δ*ler* transformed with pSE380 |
| PA20 Δ*ler*+pSE380::*pchA* | PA20Δ*ler*+ *pchA* | PA20 Δ*ler* transformed with pSE380 carrying *pchA*, *B*, *C*, *D*, *E* or *grlA* genes |
| PA20 Δ*ler*+pSE380::*pchB* | PA20Δ*ler*+*pchB* |  |
| PA20 Δ*ler*+pSE380::*pchC* | PA20Δ*ler*+*pchC* |  |
| PA20 Δ*ler*+pSE380::*pchD* | PA20Δ*ler*+*pchD* |  |
| PA20 Δ*ler*+pSE380::*pchE* | PA20Δ*ler*+*pchE* |  |
| PA20 Δ*ler*+pSE380::*grlA* | PA20Δ*ler*+*grlA* |  |
| PA20 Δ*espA*+pSE380 | PA20Δ*espA*+pSE380 | PA20 Δ*espA* transformed with pSE380 |
| PA20 Δ*espA*+pSE380::*pchA* | PA20Δ*espA*+ *pchA* | PA20 Δ*espA* transformed with pSE380 carrying *pchA*, *B*, *C*, *D*, *E* or *grlA* genes |
| PA20 Δ*espA*+pSE380::*pchB* | PA20Δ*espA*+ *pchB* |  |
| PA20 Δ*espA*+pSE380::*pchC* | PA20Δ*espA*+ *pchC* |  |
| PA20 Δ*espA*+pSE380::*pchD* | PA20Δ*espA*+ *pchD* |  |
| PA20 Δ*espA*+pSE380::*pchE* | PA20Δ*espA*+ *pchE* |  |
| PA20 Δ*espA*+pSE380::*grlA* | PA20Δ*espA*+ *grlA* |  |
| PA20 Δ*csgBA*+pSE380 | PA20Δ*csgBA*+pSE380 | PA20 Δ*csgBA* transformed with pSE380 |
| PA20 Δ*csgBA*+pSE380::*pchA* | PA20Δ*csgBA*+ *pchA* | PA20 Δ*csgBA* transformed with pSE380 carrying *pchA*, *B*, *C*, *D*, *E* or *grlA* genes |
| PA20 Δ*csgBA*+pSE380::*pchB* | PA20Δ*csgBA*+ *pchB* |  |
| PA20 Δ*csgBA*+pSE380::*pchC* | PA20Δ*csgBA*+*pchC* |  |
| PA20 Δ*csgBA*+pSE380::*pchD* | PA20Δ*csgBA*+*pchD* |  |
| PA20 Δ*csgBA*+pSE380::*pchE* | PA20Δ*csgBA*+*pchE* |  |
| PA20 Δ*csgBA*+pSE380::*grlA* | PA20Δ*csgBA*+*grlA* |  |
| 20R2R | 20R2R | PA20 missing Sp15 prophage in *mlrA* gene |
| 20R2R Δ*ler* | 20R2RΔ*ler* | 20R2R deletion of *ler*, *grlA*, *pchA*, *phcB*, *pchC* or *pchE* genes |
| 20R2R Δ*grlA* | 20R2RΔ*grlA* |  |
| 20R2R Δ*pchA* | 20R2RΔ*pchA* |  |
| 20R2R Δ*pchB* | 20R2RΔ*pchB* |  |
| 20R2R Δ*pchC* | 20R2RΔ*pchC* |  |
| 20R2R Δ*pchE* | 20R2RΔ*pchE* |  |
| 20R2R+pSE380 | 20R2R+pSE380 | 20R2R transformed with pSE380 |
| 20R2R+pSE380::*pchA* | 20R2R+*pchA* | 20R2R transformed with pSE380 carrying *pchA*, *B*, *C*, *D*, *E* or *grlA* genes |
| 20R2R+pSE380::*pchB* | 20R2R+*pchB* |  |
| 20R2R+pSE380::*pchC* | 20R2R+*pchC* |  |
| 20R2R+pSE380::*pchD* | 20R2R+*pchD* |  |
| 20R2R+pSE380::*pchE* | 20R2R+*pchE* |  |
| 20R2R+pSE380::*grlA* | 20R2R+*grlA* |  |
| 20R2R Δ*ler*+pSE380 | 20R2RΔ*ler*+pSE380 | 20R2R Δ*ler* transformed with pSE380 |
| 20R2R Δ*ler*+pSE380::*pchA* | 20R2RΔ*ler*+*pchA* | 20R2R Δ*ler* transformed with pSE380 carrying *pchA*, *B*, *C*, *D*, *E* or *grlA* genes |
| 20R2R Δ*ler*+pSE380::*pchB* | 20R2RΔ*ler*+*pchB* |  |
| 20R2R Δ*ler*+pSE380::*pchC* | 20R2RΔ*ler*+*pchC* |  |
| 20R2R Δ*ler*+pSE380::*pchD* | 20R2RΔ*ler*+*pchD* |  |
| 20R2R Δ*ler*+pSE380::*pchE* | 20R2RΔ*ler*+*pchE* |  |
| 20R2R Δ*ler*+pSE380::*grlA* | 20R2RΔ*ler*+*grlA* |  |
| 20R2R Δ*espA*+pSE380 | 20R2RΔ*espA*+pSE380 | 20R2R Δ*espA* transformed with pSE380 |
| 20R2R Δ*espA*+pSE380::*pchA* | 20R2RΔ*espA*+*pchA* | 20R2R Δ*espA* transformed with pSE380 carrying *pchA*, *B*, *C*, *D*, *E* or *grlA* genes |
| 20R2R Δ*espA*+pSE380::*pchB* | 20R2RΔ*espA*+*pchB* |  |
| 20R2R Δ*espA*+pSE380::*pchC* | 20R2RΔ*espA*+*pchC* |  |
| 20R2R Δ*espA*+pSE380::*pchD* | 20R2RΔ*espA*+*pchD* |  |
| 20R2R Δ*espA*+pSE380::*pchE* | 20R2RΔ*espA*+*pchE* |  |
| 20R2R Δ*espA*+pSE380::*grlA* | 20R2RΔ*espA*+*grlA* |  |
| 20R2R Δ*csgBA*+pSE380 | 20R2RΔ*csgBA*+pSE380 | 20R2R Δ*csgBA* transformed with pSE380 |
| 20R2R Δ*csgBA*+pSE380::*pchA* | 20R2RΔ*csgBA*+*pchA* | 20R2R Δ*csgBA* transformed with pSE380 carrying *pchA*, *B*, *C*, *D*, *E* or *grlA* genes |
| 20R2R Δ*csgBA*+pSE380::*pchB* | 20R2RΔ*csgBA*+*pchB* |  |
| 20R2R Δ*csgBA*+pSE380::*pchC* | 20R2RΔ*csgBA*+*pchC* |  |
| 20R2R Δ*csgBA*+pSE380::*pchD* | 20R2RΔ*csgBA*+*pchD* |  |
| 20R2R Δ*csgBA*+pSE380::*pchE* | 20R2RΔ*csgBA*+*pchE* |  |
| 20R2R Δ*csgBA*+pSE380::*grlA* | 20R2RΔ*csgBA*+*grlA* |  |
| 43894OR | OR | ATCC 43894 strain with spontaneous single nucleotide mutation in the *csgDEFG* -10 promoter region conferring temperature-independent elevated curli production |

**Supplementary Table 2.** Primers used in this study.

| **Primer** | **Sequence** | **Purpose** |
| --- | --- | --- |
| pchEred50F | aggcgatgacccttacggtgacggtattccgttctgagggggtggcgatgaattaaccctcactaaagggcg | *pchE* deletion |
| pchEred50R | ccataccgtttcaactggtgcaaaaaaagccggatttctccggctgttgattataatacgactcactatagggctc | *pchE* deletion |
| grlAred60F | taattttatgtatgttttttatgtcgatttatttatcaaataaaaagaatatggaaaatgaattaaccctcactaaagggcg | *glrA* deletion |
| grlAred60R | cccttaaatatagctttatttttattcttctataaaatatactcaaaaaattacgtctaattataatacgactcactatagggctc | *glrA* deletion |
| LERred50F | tatttcatcttccagctcagttatcgttatcatttaattatttcatgttaaattaaccctcactaaagggcg | *ler* deletion |
| LERred50R | tgttggtccttcctgataaggtcgctaatagcttaaaatattaaagcatgtaatacgactcactatagggctc | *ler* deletion |
| pchCred50F | ataatcggaggtcacttatgctacatgatcacctggcagaatgtctggagaattaaccctcactaaagggcg | *pchC* deletion |
| pchB/Cred50R | agtcgctttttcttatggtaacaggcaataacgctctcagatattttttataatacgactcactatagggctc | *pchB/C* deletion |
| pchBred50F | tttattgttgtcttattaaggacggtaaattcaggatggcagtctgtagaaattaaccctcactaaagggcg | *pchB* deletion |
| pchAred50F | ggaaggtaaattcaggatggcagtctgtagataatcggaggtcacttatgaattaaccctcactaaagggcg | *pchA* deletion |
| pchAred50R | gacaaacacgaccagagcacctgttcagacaggtttacttaaacgacttataatacgactcactatagggctc | *pchA* deletion |
| EspAred50F | gttacagacagggtatcgttatttacgttaagcatagttatctccggttaaattaaccctcactaaagggcg | *espA* deletion |
| EspAred50R | caataatttttttgttttcctgagaaaaattatcaagaggtatatagatgtaatacgactcactatagggctc | *espA* deletion |
| pchABC380R | aacgagctcttagcatttttttgaccgcgcgtttc | PchA/B/C expression |
| pchA380F | aagccatggataatcggaggtcacttatgctacatgatcacg | PchA expression |
| pchB380F | aagccatggataaacggaggttacttatgctacatgatcacc | PchB expression |
| pchC380F | aagccatggataatcggaggtcacttatgctacatgatcacc | PchC expression |
| pchD380F | aagccatggaaagagtaccccggaagttgtg | PchD expression |
| pchD380R | aacgagctcctgtttgccattccttgcttcatc | PchD expression |
| pchE380F | aagccatggtgacggtattccgttctgag | PchE expression |
| pchE380F | aacgagctccggctgttgattagctgtctgg | PchE expression |
| RT-CsgDF | tggacgatatctcttcaggctc | RT-PCR |
| RT-CsgDR | cgcggtacgggtaatcttcag | RT-PCR |
| RT-CsgAF | gagctgaacatttaccagtacgg | RT-PCR |
| RT-CsgAR | atcgattgagctgtcatctgagc | RT-PCR |

**Supplementary Table 3.** *P*-values referred to **Figure 2** (Effect of HTR regulator expression on biofilm formation). Data were analyzed by pairwise t-tests without assuming equal variance and adjusting the resulting *P*-values with the Benjamini-Hochberg procedure.

| **20R2R** | **+380** | **Δ*ler*+380** | **Δ***espA***+380** | **+*pchA*** | **Δ*ler*+*pchA*** | **Δ*espA*+*pchA*** | **+*pchB*** | **Δ*ler*+*pchB*** | **Δ*espA*+*pchB*** | **+*pchC*** | **Δ*ler*+*pchC*** | **Δ*espA*+*pchC*** | **+*pchD*** | **Δ*ler*+*pchD*** | **Δ*espA*+*pchD*** | **+*pchE*** | **Δ*ler*+*pchE*** | **Δ*espA*+*pchE*** | **+*grlA*** | **Δ*ler*+*grlA*** |
| --- | --- | --- | --- | --- | --- | --- | --- | --- | --- | --- | --- | --- | --- | --- | --- | --- | --- | --- | --- | --- |
| **Δ*ler*+380** | 0.057 | - | - | - | - | - | - | - | - | - | - | - | - | - | - | - | - | - | - | - |
| **Δ*espA*+380** | 0.718 | 0.022 | - | - | - | - | - | - | - | - | - | - | - | - | - | - | - | - | - | - |
| **+*pchA*** | 1e-05 | 2e-09 | 5e-05 | - | - | - | - | - | - | - | - | - | - | - | - | - | - | - | - | - |
| **Δ*ler*+*pchA*** | 0.021 | 0.703 | 0.007 | 3e-10 | - | - | - | - | - | - | - | - | - | - | - | - | - | - | - | - |
| **Δ*espA*+*pchA*** | 7e-04 | 3e-07 | 0.002 | 0.293 | 5e-08 | - | - | - | - | - | - | - | - | - | - | - | - | - | - | - |
| **+*pchB*** | 0.072 | 4e-04 | 0.143 | 0.018 | 1e-04 | 0.174 | - | - | - | - | - | - | - | - | - | - | - | - | - | - |
| **Δ*ler*+*pchB*** | 1e-05 | 0.015 | 3e-06 | 4e-15 | 0.042 | 1e-12 | 2e-08 | - | - | - | - | - | - | - | - | - | - | - | - | - |
| **Δ*espA*+*pchB*** | 8e-06 | 1e-09 | 4e-05 | 0.949 | 1e-10 | 0.309 | 0.017 | 1e-15 | - | - | - | - | - | - | - | - | - | - | - | - |
| **+*pchC*** | 0.828 | 0.140 | 0.598 | 3e-05 | 0.066 | 0.001 | 0.066 | 2e-04 | 2e-05 | - | - | - | - | - | - | - | - | - | - | - |
| **Δ*ler*+*pchC*** | 0.010 | 0.528 | 0.003 | 8e-11 | 0.797 | 2e-08 | 4e-05 | 0.077 | 3e-11 | 0.038 | - | - | - | - | - | - | - | - | - | - |
| **Δ*espA*+*pchC*** | 0.069 | 3e-04 | 0.142 | 0.013 | 7e-05 | 0.149 | 0.968 | 9e-09 | 0.013 | 0.064 | 3e-05 | - | - | - | - | - | - | - | - | - |
| **+*pchD*** | 4e-04 | 2e-07 | 0.001 | 0.434 | 4e-08 | 0.819 | 0.124 | 1e-12 | 0.459 | 6e-04 | 1e-08 | 0.104 | - | - | - | - | - | - | - | - |
| **Δ*ler*+*pchD*** | 0.128 | 5e-04 | 0.253 | 0.002 | 1e-04 | 0.053 | 0.690 | 8e-09 | 0.002 | 0.114 | 4e-05 | 0.703 | 0.034 | - | - | - | - | - | - | - |
| **Δ*espA*+*pchD*** | 0.003 | 3e-06 | 0.010 | 0.142 | 5e-07 | 0.699 | 0.344 | 2e-11 | 0.147 | 0.004 | 1e-07 | 0.312 | 0.548 | 0.136 | - | - | - | - | - | - |
| **+*pchE*** | 3e-12 | <2e-16 | 2e-11 | 0.009 | <2e-16 | 2e-04 | 8e-07 | <2e-16 | 0.006 | 4e-11 | <2e-16 | 3e-07 | 6e-04 | 8e-09 | 3e-05 | - | - | - | - | - |
| **Δ*ler*+*pchE*** | <2e-16 | <2e-16 | <2e-16 | 4e-05 | <2e-16 | 9e-08 | 9e-11 | <2e-16 | 2e-05 | 3e-16 | <2e-16 | 2e-11 | 8e-07 | 4e-14 | 9e-09 | 0.226 | - | - | - | - |
| **Δ*espA*+*pchE*** | <2e-16 | <2e-16 | <2e-16 | 1e-05 | <2e-16 | 3e-08 | 4e-11 | <2e-16 | 5e-06 | 3e-16 | <2e-16 | 1e-11 | 2e-07 | 4e-14 | 3e-09 | 0.090 | 0.560 | - | - | - |
| **+*grlA*** | 7e-05 | 2e-08 | 3e-04 | 0.712 | 3e-09 | 0.522 | 0.048 | 7e-14 | 0.749 | 1e-04 | 9e-10 | 0.038 | 0.690 | 0.009 | 0.292 | 0.003 | 7e-06 | 2e-06 | - | - |
| **Δ*ler*+*grlA*** | 1e-12 | 2e-07 | 1e-11 | 0.026 | <2e-16 | 5e-04 | 2e-06 | <2e-16 | 0.017 | 3e-11 | <2e-16 | 6e-07 | 0.002 | 9e-09 | 8e-05 | 0.553 | 0.039 | 0.011 | 0.008 | - |
| **Δ*espA*+*glrA*** | 5e-04 | <2e-16 | 0.002 | 0.325 | 4e-08 | 0.948 | 0.152 | 9e-13 | 0.343 | 8e-04 | 1e-08 | 0.133 | 0.870 | 0.044 | 0.651 | 2e-04 | 1e-07 | 4e-08 | 0.560 | 6e-04 |

**Supplementary Figure 1.** CR affinity of PA20 and PA20Δ*pchE* independent trials spotted on TA and incubated at 30°C and 37°C for 24 and 48 h.


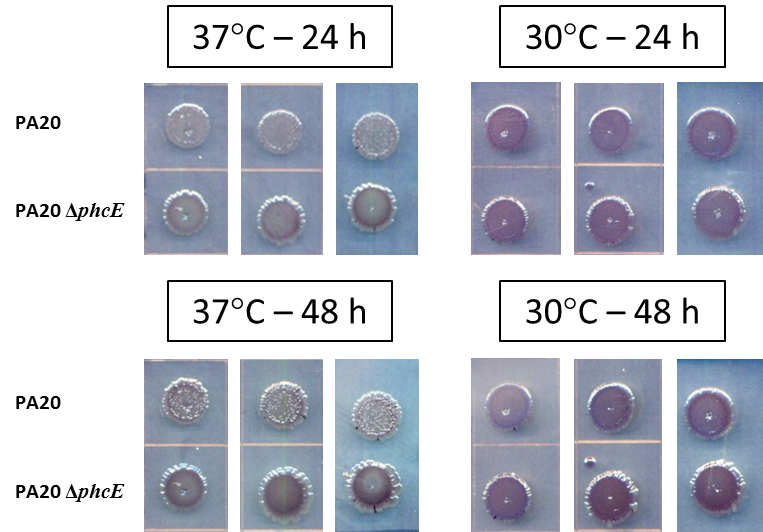

Supplement: Supplementary file 1 [file Table_1.docx]
